# Experimental Verification of Modeled Thermal Distribution Produced by a Piston Source in Physiotherapy Ultrasound

**DOI:** 10.1155/2016/5484735

**Published:** 2016-11-23

**Authors:** M. I. Gutierrez, S. A. Lopez-Haro, A. Vera, L. Leija

**Affiliations:** ^1^CONACYT, Instituto Nacional de Rehabilitación, Subdirección de Investigación Tecnológica, Calz. México Xochimilco, No. 289, Col. Arenal de Guadalupe, Mexico City, Mexico; ^2^Centro de Investigación y de Estudios Avanzados del IPN (Cinvestav-IPN), Electrical Engineering Department, Bioelectronics Section, Av. Instituto Politécnico Nacional 2508, Col. San Pedro Zacatenco, Del. Gustavo A. Madero, Mexico City, Mexico

## Abstract

*Objectives*. To present a quantitative comparison of thermal patterns produced by the piston-in-a-baffle approach with those generated by a physiotherapy ultrasonic device and to show the dependency among thermal patterns and acoustic intensity distributions.* Methods*. The finite element (FE) method was used to model an ideal acoustic field and the produced thermal pattern to be compared with the experimental acoustic and temperature distributions produced by a real ultrasonic applicator. A thermal model using the measured acoustic profile as input is also presented for comparison. Temperature measurements were carried out with thermocouples inserted in muscle phantom. The insertion place of thermocouples was monitored with ultrasound imaging.* Results*. Modeled and measured thermal profiles were compared within the first 10 cm of depth. The ideal acoustic field did not adequately represent the measured field having different temperature profiles (errors 10% to 20%). Experimental field was concentrated near the transducer producing a region with higher temperatures, while the modeled ideal temperature was linearly distributed along the depth. The error was reduced to 7% when introducing the measured acoustic field as the input variable in the FE temperature modeling.* Conclusions*. Temperature distributions are strongly related to the acoustic field distributions.

## 1. Introduction

In ultrasonic physiotherapy, it is common to employ different sizes of transducers for treating diverse areas of the body. Each transducer produces a unique acoustic field pattern due to the particular vibration conditions of the piezoelectric disks related to the variability of transducers manufacturing process [1–3]. These variations in the radiation pattern should influence the temperature distributions and could be the cause of unexpected acoustic radiations [1]. Even though many efforts have been made in order to establish standards to increase the effectiveness of the treatment [4–7], other important aspects have not yet been considered, such as the effects of intrinsic focusing related to the transducer manufacturing [3], the effect of the transducer front-face heating due to ineffective ultrasound emission [6, 8], and the relation between radiation patterns and temperature distribution (exposed in this paper). Although isolated evidence points out the potential of using ultrasound in the treatment of many illnesses [9–12], in practice, conclusive physiological improvement when using ultrasound in controlled trials has not been accomplished [13–17]. This situation could be the consequence of the subjective variables involved in the common practice of this therapy and the need to introduce more parameters in order to standardize this application [18]. We must find better parameters (probably device dependent) that could potentially affect the therapy outcome in order to completely standardize the technique and to achieve reproducible clinical results [18].

In clinical practice, the therapeutic ultrasound is commonly applied following a dynamic treatment protocol in which the transducer does not remain static but continuously moves around the region where the lesion is located. Although dynamic protocols are often recommended to avoid hot spots, this strategy incorporates certain subjectivities to the therapy that could influence the overall efficacy. For instance, the definitive protocol for applying the therapy is often chosen by therapists based on their own experience, from which they decide the size of the treatment area (e.g., twice the transducer emitting surface) [19] and the treatment dose [20]. The scanning speed also depends on the therapist, but it has been reported by Weaver et al. [21] that the changes in average heating using different scanning speed within a specific treatment area were not significant. Grey in 2003 [19] found significant variations in the scanning speed and important differences in the dynamic strategies of therapy application when analyzing the transducer movement patterns produced by different therapists. Demchak and Stone [22] reported a difference in deep heating in human subjects when using clinically observed treatment parameters and the recommended treatment parameters that research suggested. The local recorded doses were inconsistent with those theoretically expected, which make us hypothesize that these differences could influence the gotten temperature in tissues. In this work, we are interested in reducing the source of subjectivities by proposing a different method for therapy application, in which the therapist does not interfere directly. Although studies of dynamic protocols are not discarded for future work, these would be carried out after determining the feasibility of improving the static application outcomes.

In literature, measurements of temperature distributions produced by physiotherapy ultrasound have been reported in both real tissues and tissue-mimicking materials (phantoms). From these publications, a small number were related to produce accurate models of acoustic field for the planar radiators used in this application [1, 23]. Furthermore, when the acoustic field was modeled, it usually referred to a piston source [24, 25] and few particular efforts were made to demonstrate the validity of this assumption. For instance, Gélat et al. [24] presented the aperture method to determine the effective radiating area of physiotherapy transducers formulated by assuming the transducer as a plane-piston. Although the validity of the method is not questioned, simplifying the transducer as a piston source is not congruent with the measured fields of this kind of radiators [1, 2, 23]. Lu et al. [25] developed a method for numerically approximating the acoustic field of hyperthermia devices in multilayer media. They also assumed the radiator as a piston source and they validated their numerical results with the Rayleigh integral. On the basis of the ideal vibrating piston, Grey [19] calculated the distribution of superficial energy that resulted from therapy applications by 22 therapists; however, Grey did not present the validation of either the resulting heating model or the simplified acoustic model. Recently, Miller et al. [26] measured* in vivo *temperature distributions generated by therapeutic ultrasound in human* triceps surae* muscle group during a dynamical treatment protocol on a region of two times the size of the transducer head. Their results indicated that the produced thermal increments were not uniform in the treatment area and among individuals, and they concluded that these differences could be related to some variables they could not control in the experiments, as the thermocouple final insertion, the size of treatment area, and so forth. However, they did not provide any theoretical model to validate the results or demonstrate their hypothesis. Following a thorough analysis of the state-of-the-art, we concluded that few efforts have been made to evaluate the capability of current acoustic field models to produce thermal distributions congruent with measurements.

Studies on hyperthermia therapies, specifically in physiotherapy applications, seldom consider realistic acoustic field patterns and their effects in thermal distributions. When modeling the acoustic field, the most used approach in ultrasonic physiotherapy literature is the piston-in-a-baffle (uniform vibrating aperture into an infinite rigid and no-vibrating baffle), which creates a characteristic diffraction pattern in the near-field zone. Moreover, when temperature profiles of physiotherapy applicators are presented, they commonly referred to the ideal acoustic field and no analysis of the importance of correctly representing the real acoustic field is made. In this paper, the thermal pattern produced by the piston-in-a-baffle model is compared to experimental measurements to verify if this ideal field accurately allows the representation of realistic temperature distributions; additionally, another modeling approach is also used for comparison in order to determine if using the real acoustic profile permits an improvement of the modeled thermal distribution. For this purpose, a finite element (FE) model of ultrasonic heating produced by a theoretical piston is compared with the heating produced by a real physiotherapy ultrasound applicator; the second approach using the measured acoustic field to feed the FE model and determine the heating is also shown. For the experiments and models, a static therapy protocol is used since in this modality the dosage is directly correlated to treatment time while presenting few effects of thermal dissipation by conduction [19]. Additionally, this approach eliminates the need to consider therapist-dependent variables, such as the scanning speed of the applicator and the irregular size of the treated area. The results of this work will allow a better understanding of these acoustic fields and the thermal effects they produce to eventually improve the efficacy of ultrasound based physiotherapy treatment.

## 2. Materials and Methods

The models were developed in a workstation with 64 GB of RAM and 8-core processor of 3.00 GHz. The experiments were performed using a physiotherapy ultrasound device radiating to a muscle-mimicking material (*phantom*). The phantom, with a cubic shape of 10 cm per side, emulated the acoustic properties of muscular tissue and was made of a water-based mixture of agarose, graphite, and glycerin. The physiotherapy ultrasonic device comprised a special-designed homemade RF amplifier and a commercial transducer of 1 MHz and 10 cm^2^ of nominal radiating area (Model 7310, Mettler Electronics Corp., USA).

### 2.1. Determination of Phantom Acoustic Properties

The phantom acoustic properties used in the models were determined by measurements. The acoustic attenuation was obtained with the Ping He formulation [27]. The phantom was split into 10 equal sections of 1 cm of thickness, and the attenuation of each section was measured. We used two wideband transducers of 3.5 MHz (Panametrics V383-SU, Olympus, USA) separated at fixed certain distance (not required for calculation), in which a transducer was used as transceiver and the other as receiver. The echo signals were amplified by a homemade wideband amplifier, and the signals were recorded by an oscilloscope (Wave Runner 6000A, LeCroy, USA) at 50 MHz of bandwidth. The transducers were immersed in distilled degassed water at 37°C controlled by a thermostatic bath (RCTB 3000, OMEGA Engineering, Inc., USA); the water has a negligible attenuation coefficient at the used frequencies [28]. Two ultrasound pulses were required for this technique, specifically, a first reference pulse for the wave traveling in water and a second pulse of the wave passing through the phantom.

**Figure 1:**
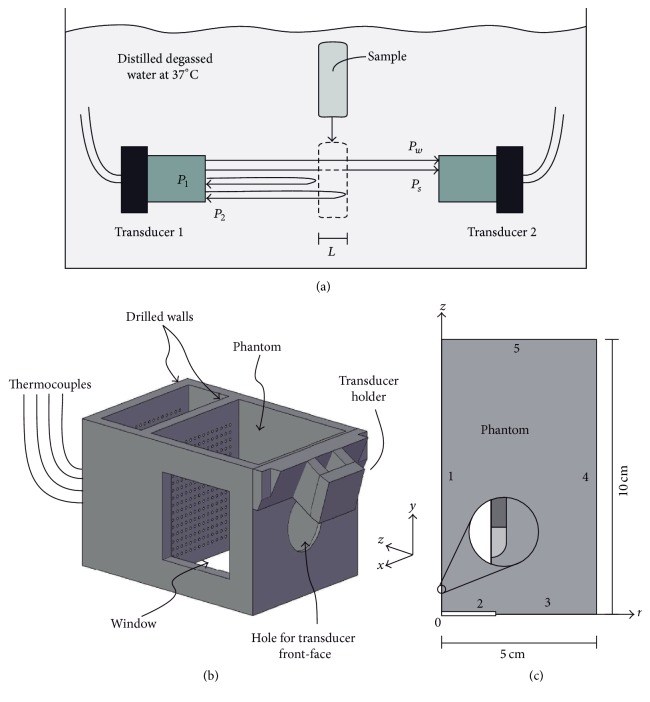
(a) Setup to measure the phantom acoustic attenuation and wave velocity. (b) Container used for the measurements. The transducer holder is dismountable to fasten other transducers of different sizes. (c) Geometry for the computational modeling; the zoom of thermocouple tip just referred to the simulations for determining the effect of thermocouple insertion in acoustic and thermal distributions.

Phantom thickness of each section was determined with ultrasound using the reference pulse *P*_*w*_, the sample pulse *P*_*s*_, and the reflected echoes produced by the interfaces phantom-water (shown in Figure 1(a) as *P*_1_ and *P*_2_) assuming *c*_*w*_ = 1523.61 m/s at 37°C [29]. Finally, the attenuation was calculated using the phantom thickness and the amplitude and phase spectra of the received pulses [27]. The speed of sound in the phantom was determined with the same setup by using the pulses produced at the phantom-water interfaces, *P*_1_ and *P*_2_.

### 2.2. Characterization of Physiotherapy Transducer

The acoustical characterization of the physiotherapy transducer was carried out by using a 3D scanning automated system (Onda Corp, USA) and a wideband needle hydrophone (SEA mod. PVDF-Z44-0400, USA) with effective aperture of 0.4 mm and sensitivity of −260 dB that referred to 1 V/*μ*Pa at 1 MHz. Both the transducer and the hydrophone were immersed in distilled degassed water to avoid bubble formation and to neglect attenuation. The measurements were made point-by-point spaced 1 mm. The transducer was excited with tone bursts of 1 MHz by using an RF amplifier card TB-1000 (Matec Instruments, USA). The hydrophone was centered before the measurements [30] to have an equally distributed radiating energy of the emission at 3 mm from the transducer. This center location was confirmed at the last maximum peak of the beam (255 mm from the transducer) with a deviation smaller than 2 mm in the *x*-axis and 1 mm in the *y*-axis. The acquired data were recorded by a computer during the process. The electrical excitation was made with 20 sinusoidal cycles and 13 ms repetition rate for simulating nearly a continuous emission of the transducer and to let the system dissipate the energy before the next measurement.

*xy* planes were recorded at different *z* positions to determine the transducer properties with reference to IEC-61689; finally, an *xz* plane (at *y* = 0) was recorded to obtain the complete acoustic field of the transducer. The transducer effective radiating area (*A*_ER_) was determined in accordance with the standard IEC-61689 [4]. The ultrasonic radiated power *P*_*R*_ emitted by the device was measured with a radiation force balance (UPM-DT-100N, Ohmic Instruments Company, USA) and this power was related to the effective acoustic intensity *I* by [7]

(1) $$\begin{array}{c} I=\frac{P_{R}}{A_{ER}}. \end{array}$$

### 2.3. Experiment Setup for Ultrasound Heating

Experiments were carried out by using a homemade container that held the transducer and the phantom and allowed guiding the thermocouples during insertion (shown in Figure 1(b)). The container had two lateral windows to monitor the thermocouple insertion with an ultrasound imaging device. The phantom and the container were immersed in distilled degassed water at 37°C regulated with a thermostatic bath (RCTB 3000, OMEGA Engineering, Inc., USA) to emulate body temperature. The center of the transducer emission was chosen as the origin of the experimental Cartesian coordinate system, then the transducer's emitting surface was placed on the *xy*-plane at *z* = 0, and the direction of wave propagation was along the *z*-direction.

Thermal maps were determined in transient state using 11 thermocouples inserted at the same depth. The thermocouples were displaced (by insertion) into the phantom to record transient temperature at nine different depths. The insertion started at *z* = 9 cm and finished at *z* = 1 cm with steps of 1 cm. The initial separation among the thermocouples was 0.5 cm determined with an ultrasound imaging device at *z* = 9 cm for *y* = 0; this means that at *z* = 9 cm the 11 thermocouples were positioned, respectively, at *x*_1_ = −2.5 cm, *x*_2_ = −2.0 cm, *x*_3_ = −1.5 cm,…, *x*_10_ = 2.0 cm, and *x*_11_ = 2.5 cm. The thermocouples were type K of 0.76 mm (0.03 in) of diameter, about 1/2 of therapy-ultrasound wavelength in the phantom (1.56 mm). This thermocouple size was chosen to permit the insertion with little spatial deviations and to facilitate the thermocouple detection by an ultrasound imaging device of 7 MHz. Thermocouples extraction was not chosen because the thermocouples leave routes of damage during extraction that can modify the thermal distribution at final depths due to direct destruction of the phantom. Because the thermocouples during insertion did not follow a real straight direction, the insertion route was monitored with an ultrasound scan (Prosound 6, Aloka, Japan) through the lateral window and the top of the container for postprocessing, as shown in Figure 1(b); the route was verified at the end of the experiment by splitting the phantom.

The experiment comprised a heating-cooling sequence for each of the nine *z*-depths; before the next heating-cooling sequence, the 11 thermocouples had been displaced to the next *z*-depth. The physiotherapy ultrasound device was set up at 12 W of ultrasonic radiated power in continuous emission; this power was verified at the end of the experiments with no variation. The heating lasted 300 s and the phantom was cooled to 37°C before next measurement; cooling was recorded during 100 s. Temperature data were taken every 2 s using NI 9219 (National Instruments, USA) and a personal computer. The thermocouples were calibrated by immersion before the experiment from 20°C to 60°C at steps of 5°C with an overall uncertainty of ±0.06°C; the thermocouples presented a linear behavior in the temperature range of experiments. Thermocouple calibration was verified after the experiments with no changes.

Thermocouple artifacts due to viscoelastic heating appeared in the temperature measurements [31, 32]. This artifact occurs by the combination of media shear viscosity and vibration [31]. Viscoelastic heating produces a rapid temperature increase at the beginning of the heating, which is followed by the exponential rise due to ultrasound absorption (effect we are analyzing) and then a fast decrease when the radiation is turned off also due to viscosity. The correction of thermocouple artifact was made by means of the detection of the stable-state temperature due to shear viscosity (*T*_0_) by back-extrapolating the exponential part of the temperature increase (due to absorption) to the start of heating (at *t* = 0) [31]. Then, *T*_0_ was subtracted just from the heating section. Immediately after ultrasound was turned off, the measured temperature was affected by both the viscous heating component and the heat dissipation by thermal conduction, which makes it complicated to know the behavior of temperature at that specific moment; therefore in Figure 3, the fast decay immediately after ultrasound was turned off was just eliminated. For instance, an Nth order function could be used to determine that section's temperature by interpolation of the cooling curve and the temperature point at *t* = 300 s, but the accuracy of the result in that part of the curve would be unknown. A back-extrapolation of the cooling exponential part to the stop-of-heating (*t* = 300 s) produced a stepped variation in temperature profile at that time for innermost thermocouples, mainly those located in large temperature gradients. This variation was produced because the heat moves from regions with higher temperature towards cooler regions, and if the thermocouple was located in the pathway, the measured temperature after cessation of ultrasound can be higher than the previous. This effect produces an upward displacement of the entire cooling curve and the back-extrapolation does not match the temperature reached at *t* = 300 s. Another possible method to correct the measurements, used by Nell and Myers [32], was to measure the “thermocouple artifact” in nonabsorbing media and subtracting it from the measurements. However, this procedure was inadequate for this work because the artifact is pressure dependent, which means that it should be measured at each spatial point of the acoustic field in a nonabsorbing phantom, and that same spatial point should be registered in the muscle phantom; in our experiments, it was not possible for the thermocouples to follow the same trajectories in both phantoms.

### 2.4. Acoustic Field Model

A first FE model for axisymmetric acoustic field during uniform acceleration distribution on the transducer surface was developed. The main objective of this approach was to obtain a computational model for radiation pattern by using the approach of the piston-in-a-baffle in stationary state during harmonic operation. The model operation conditions were based on the experiments in which the device emitted a continuous stationary harmonic radiation with a negligible start up time of 100 ms. A second approach was developed to improve the results with respect to the measured data. For this, the measured acoustic field was used as the input data of the bioheat FE simulation. However, because the transducer acoustic field was measured at low power excitation in nonattenuating medium, data preprocessing was required; for this, the measured intensity field was normalized using the average intensity into the effective radiating area; then the field was attenuated based on the measured phantom attenuation and was multiplied by a factor corresponding to the acoustic power used for experiments. To clarify, we are going to call the model using the piston source model A and the model using the measured field as acoustic data model B.

The problem was considered axisymmetric with the geometry shown in Figure 1(c) (zoom in presented is related to other analyses that will be shown later). The FE model was developed based on the cylindrical coordinate system (*r*, *z*), where the transducer was located at *z* = 0 and the radiation propagated in the *z*-direction. The variable *r* in the model is related to the experimental coordinates *x* and *y* by $r=\sqrt{x^{2}+y^{2}}$. The boundaries were defined in accordance with the experimental setup. Boundary 3 was simulated as an acoustically rigid wall by setting the normal velocity at zero. Boundary 1 was the propagation axis of the problem. Boundaries 4 and 5 had the same acoustic impedance as the media (*z* = *ρc*) to avoid wave reflections, mainly at boundary 5. The FE mesh for acoustic model consisted of 80000 squared elements, that is, about six per wavelength. The transducer uniform vibration was set up at boundary 2 by using 1 MHz harmonic acceleration with constant amplitude. This acceleration was related to the acoustic intensity by using some basic relations for a plane wave that could be considered true just on the transducer surface [33]. At first, the pressure amplitude on the emitting surface was determined based on the acoustic intensity by using [34]

(2) $$\begin{array}{c} \left|\;p\;\right|=\sqrt{2\rho cI}, \end{array}$$

where |*p*| is the average pressure amplitude on the radiator surface (Pa), *ρ* is the average medium density (kg m^−3^), *c* is the speed of sound in the medium (m s^−1^), and *I* is the effective acoustic intensity (W m^−2^) obtained using (1). Then, considering a plane wave behavior on the transducer surface, the piston particle velocity *v*_0_ and the pressure on the surface *p*_0_ can be related by *p*_0_ = *ρcv*_0_ [33]. Moreover, if we also consider the harmonic operation, the transducer emission can be set up as either the uniform normal velocity or the uniform normal acceleration on the surface. The acceleration *a*_0_ on the radiator surface can be obtained by deriving the constant amplitude harmonic normal velocity *v*_0_ *e* ^*jωt*^ with respect to the time as

(3) $$\begin{array}{c} a_{0}=v_{0}\omega e^{j\left(\omega t+\pi /2\right)}=\frac{\omega }{\rho c}p_{0}e^{j\left(\omega t+\pi /2\right)}. \end{array}$$

The term *π*/2 in (3) is the phase shift between the velocity and the acceleration, and this does not affect their uniformity along the transducer surface. The acceleration of (3) can be established in terms of the intensity by substituting (2) into (3). The considerations used to derive (3) are valid just on the transducer surface, where the wave can be considered planar.

The experiments were carried out in continuous-mode radiation during stationary harmonic condition; therefore, the acoustic field was modeled in this regime. The equation of harmonic wave propagation can be given as [1, 35]

(4) $$\begin{array}{c} \nabla^{2}p+{k_{c}}^{2}p=0, \end{array}$$

where *p* is the spatial pressure and *k*_*c*_ is the complex wavenumber (*k*_*c*_ = *k* − *iμ*, where *μ* is the attenuation coefficient, *k* = 2*π*/*λ*, and *λ* is the wavelength). Based on the transducer geometry, the problem was simplified by considering axisymmetric radiation, situation in which (4) is still valid. The attenuation was assumed constant and homogeneous. The attenuated acoustic field computed with our FE model was compared with the attenuated field calculated with the Rayleigh integral with attenuation.

### 2.5. Ultrasonic Heating Model

Concerning the relation of ultrasound and temperature, the time-average heat *Q* produced in the media depends on both the intensity *I* and the pressure amplitude absorption coefficient *α*, as *Q* = 2*αI* [36]; here, the coefficient *α* is related to the pressure amplitude attenuation coefficient *μ* by *μ* = *α* + *β*, where *β* is the scattering. For this case, it was considered that all the attenuation was produced by absorption and the scattering effect was negligible. Subsequently, the temperature distribution *T* in the medium was obtained by the Pennes bioheat transfer equation with no contribution of metabolism and without blood flow, given by

(5) $$\begin{array}{c} \rho C\frac{\partial T}{\partial t}-\overset{-}{k}\nabla^{2}T=Q_{ext}, \end{array}$$

where *ρ* is the medium density (kg m^−3^), *C* is the medium heat capacity (J kg^−1^ K^−1^), $\overset{-}{k}$ is the medium thermal conductivity (W m^−1^ K^−1^), and *Q*_ext_ is the heat per volume and time (W m^−3^) produced by an external source. This external heat is related to the pressure [34] by

(6) $$\begin{array}{c} Q_{ext}\left(\;r,z\;\right)=2\alpha I\left(\;r,z\;\right)=\frac{\alpha {\left|p\left(r,z\right)\right|}^{2}}{\rho c}\;\left[\;W/m^{3}\;\right], \end{array}$$

where *c* is the speed of sound in the medium (m s^−1^) and |·| denotes the amplitude of the spatial pressure.

The stable-state harmonic acoustic field in media with homogeneous attenuation was used as the input of the heating problem employing *Q*_ext_ of (6). The coordinate system was the same as that of the FE model for the acoustic field. The boundary conditions were chosen in accordance with the experimental setup. Boundary 1 was considered as the symmetry axis, similar to that of the acoustic field model. Boundary 2 was set as thermally insulated and then $n\cdot \left(\overset{-}{k}\nabla T\right)=0$, where **n** is a normal unit vector. Boundaries 3, 4, and 5 were configured to have 37°C, which was the temperature of the water in the experiments (controlled by the thermostatic bath). The time steps for modeling the transient temperature were 10 s and the heating finished at 300 s, as in the experiments; in addition, cooling of 100 s was also included in the model.

### 2.6. Analysis of Thermocouple Acoustic Field Perturbation

The effect in the acoustic field and thermal distributions of thermocouples was analyzed with FE simulations under the same operation conditions of the models and for different thermocouples sizes that ranged from 0.1 mm to 1.6 mm of diameter (*Ø*). This effect was analyzed in contrast to the nonperturbed situation with no thermocouple inserted in the media. Regarding the zoom in Figure 1(c), a thermocouple was included along the propagation axis as it was inserted in the opposite direction of the field propagation starting from *z* = 10 cm at *r* = 0 with the tip final location at *z* = 1 cm and *r* = 0; the tip had a round shape, as with real thermocouples, and the tip boundary acoustic condition was configured as a rigid wall (zero particle velocity). The acoustic properties of the thermocouple body were those for the thermocouples cover material PFA (Perfluoroalkoxy Copolymer), namely, the PFA density *ρ*_PFA_ = 2150 [kg/m^3^], obtained from DuPont™ PFA datasheet, and the speed of sound of PFA *c*_PFA_ = 920 [m/s], calculated from [37] $c=\sqrt{y\left(1-\nu \right)/\left(\rho \left(1+\nu \right)\left(1-2\nu \right)\right)}$, where *y* and *ν* are Young's modulus and Poisson's ratio, respectively, whose values were *y* = 480 MPa and *ν* = 0.45. The mesh size was improved to 6 elements per millimeter to get about 9 elements per wavelength. The results of simulations were analyzed around the thermocouple tip to determine how the thermocouple perturbs the acoustic field and how this perturbation modifies the measured temperature. Variations in PFA acoustic properties of even 50% did not importantly modify the results; this is because we are not analyzing the field or temperature perturbation at the thermocouple body. The results of simulations were confirmed experimentally by using two different size thermocouples of Ø = 0.76 mm and Ø = 0.26 mm inserted in the graphite phantom at 1 cm from the transducer.

### 2.7. Measuring Transducer Front-Face Heating

The transducer front-face heating was measured and analyzed to determine its contribution on the obtained thermal distribution. For this, it used a cube shape agarose phantom with 6 cm per side. The transducer and the phantom were immersed in distilled degassed water at 37°C and were fixed together to avoid any relative movement. A type J wire thermocouple of 0.26 mm (0.01 in) of diameter was set between them at the center (*x* = *y* = *z* = 0) to measure the temperature produced by the transducer front-face heating. Temperature was recorded each second using NI 9219 (National Instruments, USA). The ultrasound device was configured to deliver 1.0 W/cm^2^ during 5 min to emulate the main experiments. Three independent heating-cooling sequences were acquired for this measurement.

The measured temperature rise was originated by two different heat sources, the transducer front-face heating and the ultrasound absorption; in these experiments, viscous heating was not observed. The agarose phantom does not have a negligible attenuation coefficient, so the heat produced by ultrasound should be accounted. To determine this contribution at this specific position but without the effect of transducer front-face heating, the temperature elevation by absorption was calculated with FE simulations. If we consider the heat sources affecting the measurements independent and separable, the transducer front-face heating can be obtained by subtracting the simulated temperature due to absorption from the measured temperature. Validity of our simulations was verified with the same setup using a gap between the transducer and the phantom to eliminate the effect of front-face heating (data not shown in this paper).

## 3. Results

### 3.1. Phantoms

The muscle phantom used in experiments was prepared with 10% of glycerin (to change speed of sound), 7% of graphite (to change absorption), 1.5% of agarose (to solidify the mixture), and 81.5% of distilled degassed water (as base of the phantom). The reported speed of sound and pressure attenuation coefficient for muscular tissue were 1550–1620 m/s and 4.0 Np/m, respectively [36, 38]; these properties were measured for the phantom at 37°C, with respective values of 1553 ± 12 m/s and 5.98 ± 0.46 Np/m. Both values were considered constant in simulations.

The agarose phantom used for measuring the transducer front-face heating was prepared with 1.5% of agarose and water. This phantom was planned to have low attenuation coefficient (and then low absorption to ultrasound) to make the heating by ultrasound negligible. However, real attenuation coefficient was important, and its inclusion in determining the transducer front-face heating was required. For this phantom, the measured speed of sound and attenuation coefficient were 1530 ± 15 m/s and 0.47 ± 0.08 Np/m, respectively.

### 3.2. Acoustic Field Characterization

**Figure 2:**
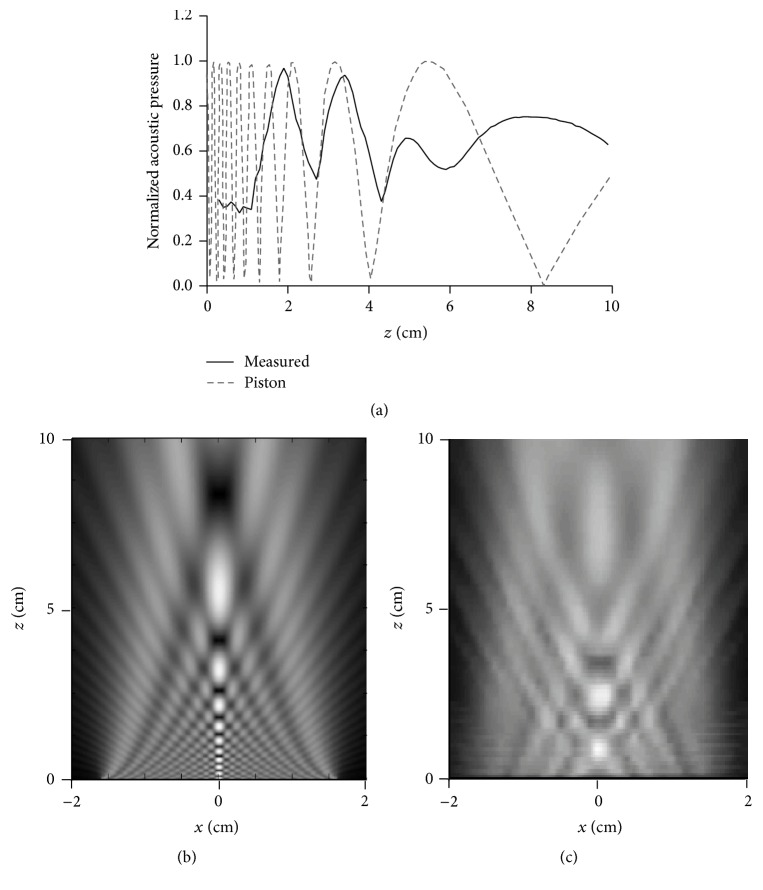
Acoustic fields of transducer and ideal piston without attenuation. (a) Normalized pressure on the propagation axis. (b) and (c) Complete acoustic field of piston and transducer, respectively (linear grayscale of normalized acoustic pressure with respect to the last maximum peak at Rayleigh distance). Comparisons between FE acoustic field and Rayleigh integral gave insignificant variations (less than 0.04% of error in certain points).

The acoustic field of the transducer was measured in distilled degassed water (attenuation-free media). The measured acoustic field was compared with the field produced by a piston, called model A (see Figure 2). We started with this ideal field because this approximation is widely used to model the radiation of this kind of transducer. As can be seen in Figure 2, the real acoustic field differs enormously from the ideal situation in the region where the therapeutic effects are produced, at the very near-field zone. Then, we decided to determine if these differences in the acoustic field patterns had an effect on the thermal distribution. Moreover, to neglect effects in the thermal profile for calculating the acoustic field with the FE method, the FE acoustic field was compared to the one obtained with the Rayleigh integral. We found negligible variations with a major difference in amplitude of 4.23% close to the radiator (before *z* = 1 cm); the overall average relative error was under 0.04%. These results suggest that we can considered valid both situations because the field calculated with the FE method is equivalent to the field obtained with the Rayleigh integral. The field with the Rayleigh integral was not included in Figure 2 because the differences are not visually evident.

The measured acoustic field was used to calculate the transducer characteristic parameters. The ultrasonic radiated power of the physiotherapy device was measured to determine the acoustic intensity to be used in the models. The measured *A*_ER_ was 8.19 cm^2^ (opposed to 10 cm^2^ indicated by the transducer manufacturer) and the measured ultrasonic power was 12 W. Using (1), the effective acoustic intensity emitted to the phantom was 1.46 W cm^−2^ and this value was used to calculate the amplitude of the acceleration for the acoustic field of model A using (2) and (3); the calculated acceleration amplitude of the radiating surface was 8.42 × 10^5^ m s^−2^. The *A*_ER_ provided the effective radiating radius to simulate the uniform emission of model A (piston radius), which was 1.61 cm. Temperature increase was obtained by using (5) and (6) considering the modeled acoustic field as the input for the heating model. For the model B, the procedure was quite different to that used for model A. Before calculating the heat, the acoustic field measured at low power excitation was normalized to permit us to adjust the intensity to that used in experiments; normalization was carried out with the average intensity close to the transducer into the *A*_ER_. The normalized intensity was multiplied by the intensity used in experiments, 1.46 W cm^−2^, and then it was artificially attenuated along *z*-axis with the exponential factor *e* ^−2*μz*^. Temperature increase was then obtained with FE using (5) and (6).

### 3.3. Postprocessing Temperature Measurements

**Figure 3:**
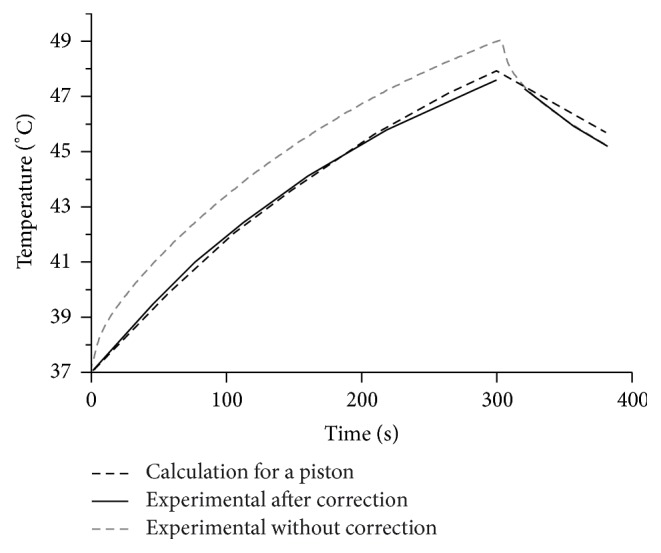
Temperature at 1 cm from the transducer radiating surface along the *z*-axis (*r* = 0) for the model and corrected and uncorrected measurements for thermocouple artifact.

 Temperature measurements were acquired in a personal computer and the postprocessing was carried out in MATLAB. Thermocouple artifact was corrected as explained above. The uncorrected temperature measurement was compared with both the corrected one and the modeled increment at the same position in Figure 3. The overall corrected measurement was further approximated to the real temperature, and it was also congruent with the model. The maximum absolute error between the corrected measurement and the model resulted in 0.36°C, meanwhile the difference between the uncorrected measurement and the model was 1.7°C.

**Figure 4:**
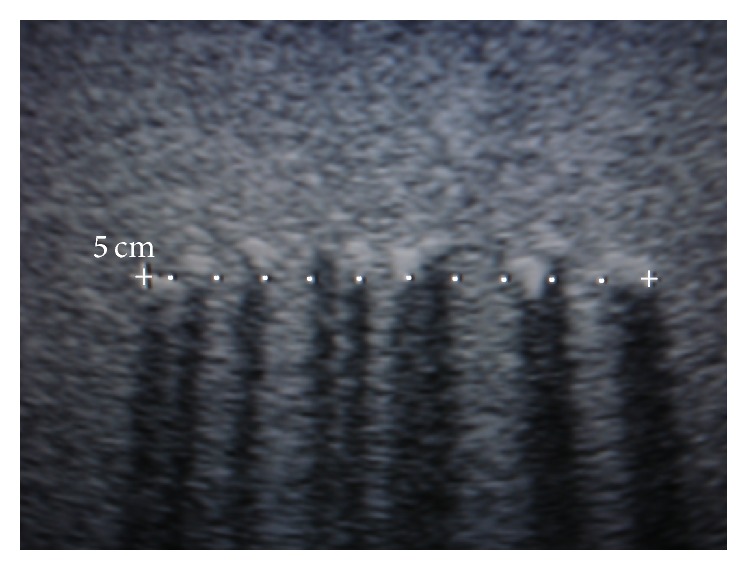
Ultrasonic image of the thermocouples into the phantom at 7 cm from the transducer; the thermocouple is shown as a clear zone followed by a dark shadow. The position of each thermocouple was determined by using these images along the insertion route, and it was verified by splitting the phantom at the end of the experiment.

After the thermocouple artifact was compensated, the thermal map inside the phantom was determined by cubic interpolating the measured temperatures; the thermocouples spatial positions used for the interpolation were obtained by following the thermocouple trajectories during insertion with the ultrasound scan; an ultrasound scan image obtained at a depth of 7 cm from the transducer radiating surface is shown in Figure 4. The complete thermal map was obtained for the *xz*-plane perpendicular to the transducer surface for *y* = 0. During insertion, thermocouples deviated from their initial position, and this deviation was accounted. The overall average on measured *y*-position and the standard deviation from the start position *y* = 0 were 1.9 mm and ±4.0 mm, respectively. In this experiment, the ratio of thermocouple diameter and the imaging-ultrasound wavelength (0.22 mm at 7 MHz) was 3.45, which was adequate to detect the thermocouples. The ratio of the thermocouple diameter (0.76 mm) and therapy-ultrasound wavelength (1.56 mm) was 0.49, which means that the thermocouple diameter represents about 1/2 of the wavelength. This thermocouple size should not appreciably affect the acoustic field at regions between the transducer surface and the thermocouple, and after that, the thermocouple would modify the acoustic field due to a shadowing effect [39]; these regions were not of our particular interest because they were located beyond the wave traveling path.

### 3.4. Thermocouple Acoustic Field Perturbation

**Figure 5:**
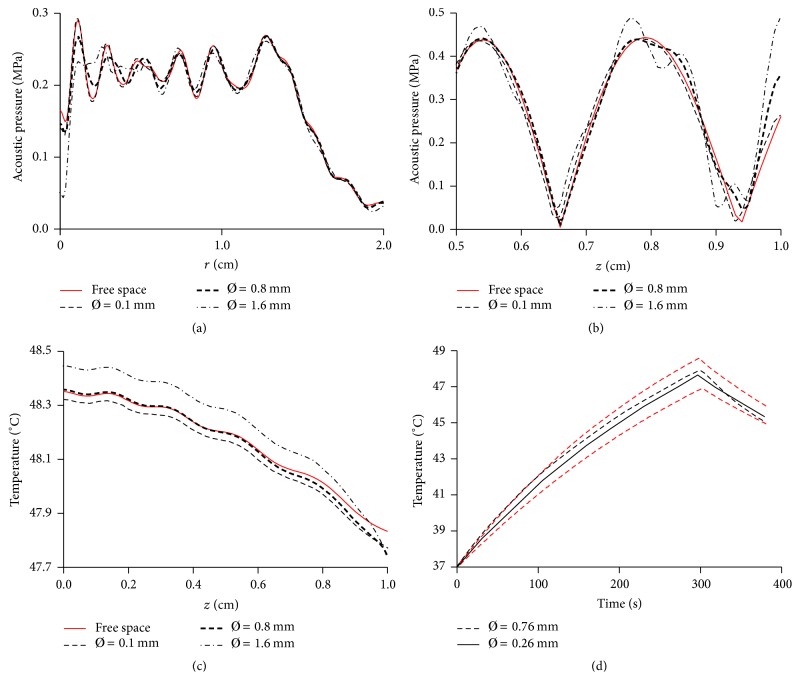
Simulations under the same experimental conditions with a thermocouple imbedded along the symmetry axis with the tip located at 1 cm from the radiator surface. (a) Radial pressure profile 1 mm in front of the thermocouple (*z* = 9 mm) for the different thermocouple diameters (*Ø*) and for the unperturbed acoustic field. (b) Acoustic pressure on the propagation axis between the thermocouple and the transducer; regions immediately in front of the transducer were omitted because important variations were not observed. (c) Simulated temperature profile on the *z*-axis between the transducer and the thermocouple (notice the scale); temperature profile of the thermocouple with Ø = 1.6 mm (one wavelength) presented larger variations than other diameters with respect to the unperturbed temperature profile. (d) Experimental temperature measured with different diameter thermocouples at 1 cm from the transducer in a graphite phantom. The red dashed-lines indicate the standard deviation (SD) of measurement using 0.26 mm diameter thermocouple; the measurements with the other thermocouples do not present important deviations and SD lines (that should appear transposing the respective dot line temperature graph) were omitted for clarity. The result of using any of two thermocouples does not appreciably differ.

As it can be observed in Figures 5(a) and 5(b), the radial and axial pressure profiles when using a 0.1 mm diameter thermocouple do not importantly differ from the unperturbed field since both lines are approximately superposed. The main difference when using this thermocouple was observed at *r* = 0 for Figure 5(a) and on the little delay in *z-*direction for Figure 5(b). Almost the same behavior can be observed in 0.8 mm diameter thermocouple, with light differences in amplitude in Figure 5(a) (maximum local difference of 12.4% at *r* = 2 mm), but keeping the shape of unperturbed field, and some distortions at 2 mm in front of the thermocouple in Figure 5(b), but with a negligible delay in *z*-direction. As positive control, the field when using a 1.6 mm diameter thermocouple was analyzed showing evident signs of perturbation in Figures 5(a) and 5(b). The modeled temperature elevations on the symmetry axis for the studied cases were plotted in Figure 5(c) (notice the expanded temperature scale). The most transcendental variation of these results was found when using a 1.6 mm diameter thermocouple, for which the temperature increment was 0.85% larger than the increment for the unperturbed situation, possibly because of acoustic field distortion. Other temperature variations shown in Figure 5 smaller than 0.25% can be considered negligible as they did not follow a predictable tendency when using different thermocouple sizes between diameters of 0.1 mm to 0.8 mm; we have concluded that this variability could be produced by the accuracy of the FE method, but it was not importantly reduced when increasing the mesh to 12 elements per wavelength.

Experiments using two different size thermocouples are shown in Figure 5(d). The curves follow the same exponential tendency and they reach a similar maximum temperature at the end of heating. The red dashed-lines indicate the standard deviation (SD) of measurements with thinner thermocouple with an averaged SD of ±0.85°C and a maximum SD of ±1.25°C; these large values of SD possibly occurred because the thermocouple positioning into the phantom at a specific location was difficult. The SD lines of the measurements with the thermocouple of 0.76 mm diameter are not shown because the SD value was small (average SD of ±0.08°C and a maximum SD of ±0.17°C) and the respective lines should appear transposing the dot line. This SD was particularly small because the rigid body of these thermocouples permitted following a more controllable insertion route.

### 3.5. Analysis of Temperature Distributions

The acoustic field and temperature distributions were determined with FE calculations. FE models were compared with experimental data to analyze their correspondence for this particular situation. Before making conclusions, the transducer front-face heating was determined to evaluate its effects in the measurements. After knowing all the variables involved in the problem (most of them well controlled), we are able to analyze the acoustic field and temperature distributions and determine their correspondence.

The transducer front-face heating was determined as explained above and the effect of this heating on the temperature measurements at different distances is now discussed. The measured temperature at *t* = 300 s between the transducer and the phantom was 39.40°C while the modeled temperature without transducer front-face heating at the same position considering the agarose phantom acoustic properties was 38.14°C. Then, the temperature increase at *t* = 300 s due to transducer front-face heating can be approximately considered as 1.26°C with an exponential tendency stating at 37°C. This temperature elevation was used to simulate the heat propagation in the graphite phantom without ultrasound (simulated temperature curves are not shown). The temperature was set in the boundary 2 of Figure 1(c), and the rest of the boundaries remained without changes as used in the other models. With this simulation, the temperature elevation at different depths produced by the transducer front-face heating can be determined. The results indicated that the temperature increase by conduction due to transducer front-face heating was 0.39°C at *z* = 1 cm, that is, 3.57% of the temperature increase in the main experiment at 1 cm. At 2 cm the effect reduces to 0.08°C, that is, 0.76% of the temperature in the main experiment. After these results, the effect of transducer front-face heating was considered negligible for our experiments. This effect could be considered important at 1 cm from the transducer, but our main conclusions are more related to other deeper distances where the effect is small.

**Figure 6:**
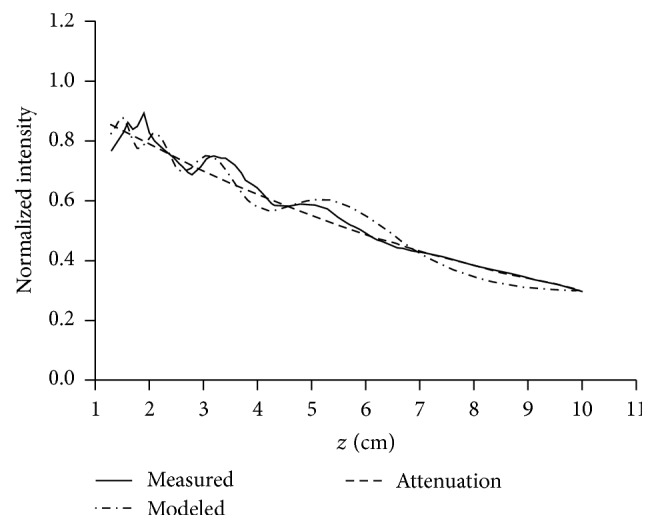
Averaged acoustic intensity at each depth of the measured (with artificial attenuation) and the modeled acoustic fields. The normalization was carried out using the averaged intensity used in experiments: 1.46 W/cm^2^. The two curves fittingly follow the theoretical curve of attenuation.

**Figure 7:**
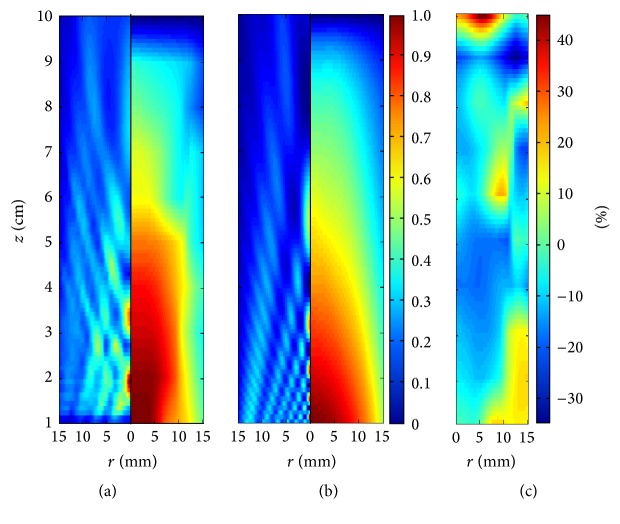
Temperature distribution (*right side of each distribution*) at 300 s versus stationary acoustic field (*left side of each distribution*): (a) experiment, (b) model A, and (c) relative errors between the measured and the modeled temperature increments. The acoustic intensities attenuate with the increase in *z*. Temperatures and intensities are normalized to use the same visualization scale.

The average acoustic intensity at each depth using all measured and modeled points was determined in order to analyze if the produced acoustic field is composed of zones with higher concentration of energy than others. Although this is an undesired behavior for this kind of transducer, these irregularities should be considered in our analysis. For instance, if this procedure were carried out for a focused transducer, the result would indicate a higher energy concentration at the focus compared to the energy concentration at other depths. The averaged intensities at each depth are shown in Figure 6, normalized using the overall average intensity of each field; the measured field was attenuated using the exponential factor *e* ^−2*μz*^. Because acoustic measurements were made in attenuation-free media, but thermal experiments and models were made in a phantom with specific attenuation, the measured intensities were attenuated to analyze the graph of Figure 6 in the conditions of temperature experiments, that is, in the mode the intensity linearly acts to produce heat (*Q* = 2*αI*) [36].  Figure 7 shows the thermal distributions obtained at 300 s for the experiment and the model in contraposition to their respective acoustic fields. Although the measured and modeled acoustic fields exposed spatial discrepancies in the intensity diffraction pattern shown in left parts of Figures 7(a) and 7(b), their average intensities of Figure 6 at each depth appropriately followed the theoretical curve of attenuation. In accordance with this similar behavior of depth-averaged acoustic intensities, the thermal patterns of model and experiments should be theoretically congruent; conversely, these thermal patterns differ when *z*-coordinate increases (right parts of Figures 7(a) and 7(b)). This is because temperature depends on heat produced spatially, as expressed with the nabla-operator in (5), and the analysis with respect to depth after simplifying the field distribution, which is often presented in the literature, is not sufficient to obtain valid thermal profiles.

**Figure 8 fig8:**
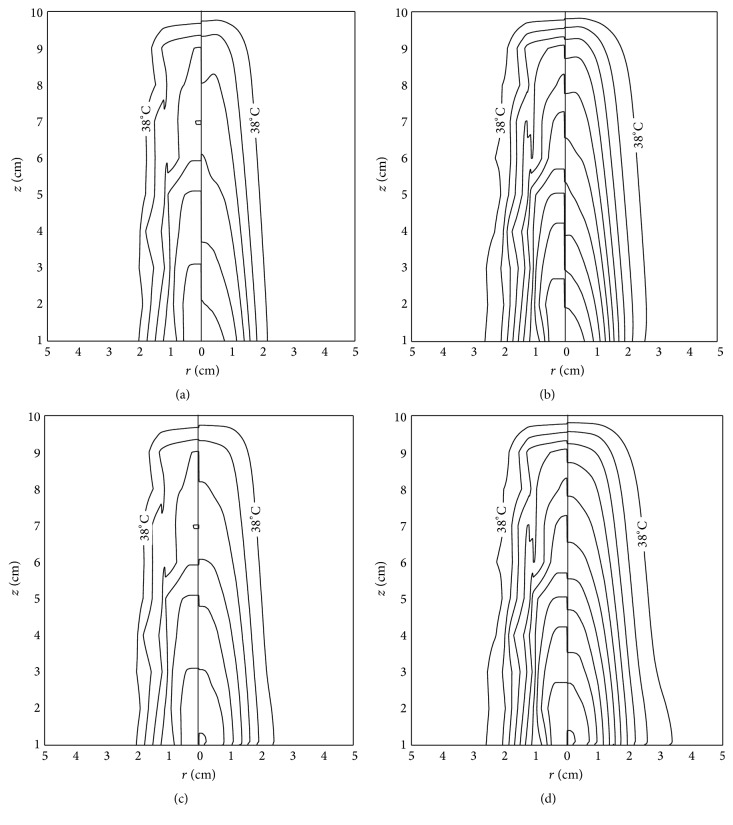
Contour plots for the experiment (*left side of each distribution*) and models (*right side of each distribution*) at two different heating times: (a) and (c) 150 s; (b) and (d) 300 s. (a) and (b) Experiment is compared with temperature produced by the piston (model A). (c) and (d) Experiment is compared with the results when modeling the heat produced by the measured acoustic field (model B). Contours from border to center: 38°C, 39°C,…,  and  47°C.

The analysis of the spatial heating effect produced by both the theoretical piston source and the real acoustic field distributions was carried out to determine the validity of this widely used theoretical approach. Returning to the graphs in Figure 3, these presented good agreement between model and measured temperatures for a specific spatial point close to the applicator with maximum absolute errors of 0.36°C. Also, the maximum temperature reached at that point was of 47.83°C in the model and 47.60°C in the measurements; the average heating rates were 0.036°C/s and 0.035°C/s for the model and measurements, respectively. However, in spite of these similitudes close to the radiator, in Figures 7 and 8, the discrepancies in the thermal distributions are evident before 6 cm of depth. While the modeled acoustic field by uniform vibrating distribution, shown in Figure 7(b), was “in average” uniform, the real acoustic field produced by a physiotherapy applicator, shown in Figure 7(a), had regions with clearly higher intensities at the first 6 cm of depth. The modeled acoustic field comprised both very high and very low intensities too close to each other, which, owing to added thermal conduction, resulted in an average heating and soft thermal increments along the depth. The region with the highest temperature increments in the experiments (the region within 0.6 of the normalized temperature plot) was obtained where the highest intensities were located in the measured acoustic field (Figure 7(a)). On the basis of the error plot shown in Figure 7(c), it can be concluded that the modeled thermal distribution is not congruent because the measured and the modeled acoustic fields differ. The relative errors of temperature increments presented in Figure 7(c) indicate discrepancies of about 15% in the first 5 cm into the very near-field zone where the strongest therapeutic effects are expected. Differences of 30% beyond 8 cm were not of particular interest because the temperature increments were significantly reduced at this depth and the relative error was magnified.

Although these results could be considered good for experiments carried out with uncontrolled influential variables (e.g., blow flow, therapist related variability, irregular multilayer tissues, and deficiencies in device calibration), the found variations should not be considered acceptable because our experiments were carried out under very controlled conditions. Therefore, the model (piston) is not an appropriate representation of the reality, and these differences should be related to another not-yet-accounted variable, as the acoustic field distribution. To test this, we used the measured acoustic field to feed the input variable (*Q*_ext_ of (5)) and get a more realistic temperature profile. For this, the acoustic field was artificially attenuated with the exponential factor *e* ^−2*μz*^ and we used the same acoustic intensity as in the experiments. These two models are compared using the thermal evolution at two specific times shown in Figure 8, for the piston produced heating (model A) and the measured field produced heating (model B). In general, the outermost contours of model A in Figures 8(a) and 8(b) accurately correspond to each other, while the inner ones do not. The central contours are strongly dependent on the acoustic field distribution as explained earlier. The contours of 47°C and 46°C in the measured part of Figure 8(b) are about 1 cm deeper than those of the model part; the same can be observed with the 43°C contour presented in Figure 8(a). This condition also appears for deeper contours, beyond 6 cm depth. However, these discordances are reduced for model B (Figures 8(c) and 8(d)). The innermost contours are closer between experiment and model B, while the outermost contours remain at the same position as for model A.

**Figure 9:**
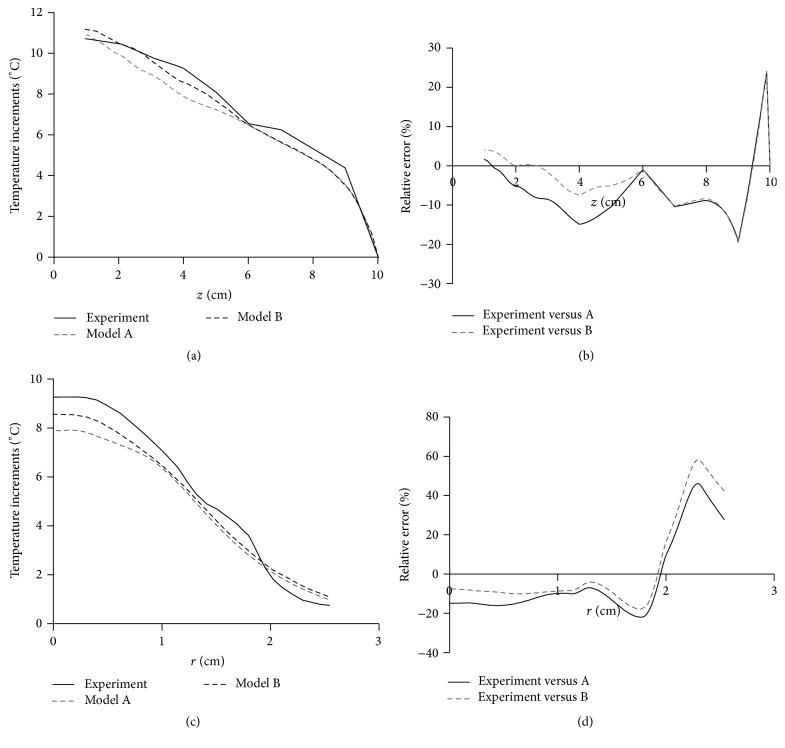
(a) Thermal profiles along *z*-direction on the acoustic propagation axis for the experiment and the models. Temperature of model A is produced by the ideal piston, and temperature of model B is produced when the measured acoustic field was used as the input excitation of the FE heating model. (b) Relative error between the measured data and both of the modeled temperatures of part (a). (c) Radial thermal profiles at 4 cm of depth for the experiment versus both of the models. (d) Relative error between the measured data and both of the modeled temperature increments of graph (c). Using the same radiation parameters, the piston (model A) produced less temperature increments than the experiment. Relative errors indicate large variations (from 10% to 20% in some regions) due to differences in the acoustic field patterns. The results are better when using the measured acoustic field as the input variable of FE simulations (model B).

The disagreements are quantitatively noticeable in Figure 9, in which the thermal profile on the propagation axis and the radial temperature at 0.4 cm are shown for both the models. At the first 6 cm of depth, the measured thermal profile differs evidently from model A while it is closer to model B, with a maximum relative error of 15% at 4 cm for model A and 7% for model B (Figures 9(b) and 9(d)). Moreover, beyond the first 6 cm, both the models gave similar results; specifically at 8 cm from the transducer, the modeled temperature was up to 10% lower than the measured one. For Figure 9(d), it should be noted that we are comparing the results by using relative errors, which were logically magnified when *r* > 2 cm due to the small temperature increments at those points; since these temperature increments were caused mainly by the thermal conduction effect and just a little contribution by the ultrasound absorption (1.1% of measured contribution of acoustic absorption when *r* = 2 cm at 4 cm), these parts were not considered in our analysis. The differences of 15% of relative error indicate about 1.4°C of temperature variation among the model and the experiment, which means, in terms of therapeutic effects, an important variation that could imply either not having any therapeutic effect [40, 41] (temperature less than 40°C [40]) or crossing the maximum temperature of 45°C suggested for physiotherapy [40]. As seen in the average intensities shown in Figure 6, these temperature differences are not directly related to the energy deposition dependent on depth, because the energy along the *z*-coordinate is similarly distributed. However, these temperature differences can be related to the energy deposition that depends on the radiation patterns. A more accurate model for acoustic field could help match the results to efficiently predict the thermal distributions in more real situations.

## 4. Discussion

The necessity of a correct validation of the effects produced during a physiotherapeutic ultrasound treatment has been pointed out by many authors in recent years [7, 14–16]. Miller et al. [15] indicated that this therapy modality has presented low level of efficacy, and at this time, the clinical benefit to the patient is unclear. More research around improving the risk-to-benefit ratio should be carried out for physiotherapeutic ultrasound. This research should incorporate studies about quality assurance and patient safety in order to protect the patient from injuries and to prevent the use of malfunctioning devices [15, 18]. Quality assurance studies would permit analyzing the device ultrasonic radiation for determining better treatment protocols to produce and standardize methods that make all the physiotherapy ultrasonic devices interchangeable. This is a clear necessity because at this moment the produced thermal effects depend on the device used [1, 3, 8, 40]. Our research group is motivated on identifying the machine and transducer critical parameters to produce an adequate acoustic field that generates the expected thermal distribution. However, there are remaining research gaps in the therapy application that should be filled to validate the technique and to explain the specific reasons to any gotten conclusion [18]. This paper was written to make researchers get involved in this topic again in order to achieve necessary improvements to this technique and make the therapy more effective and predictable.

With the presented results, it has been demonstrated that the acoustic field of a piston source does not generate a thermal distribution that completely corresponds to those obtained using real physiotherapy ultrasonic applicators. This is a major problem because the piston theory is often applied to easily model the acoustic energy into the biologic media to study the thermal distributions at different depths. Although the discordance between this ideal model and real acoustic fields is widely known, especially for the narrow-band transducers used in physiotherapy, the ideal models are continuously used in the study of this radiation without considering the error source produced by differences in the field patterns. The temperature differences of about 1.4°C found in this study, between the model and the experiment, can impact the practical therapeutic effects because of the small temperature range of physiotherapy ultrasound (from 40°C to 45°C) that determines having or not an effective treatment, or even provoking injuries to the patient [40]. Although the obtained differences are only valid for static treatment protocols under the detailed experimental conditions, we demonstrated that the acoustic field distributions do have an important effect in thermal distributions, and the piston approximation is not the adequate model for this kind of transducers. The use of a static protocol for these experiments is related to the interest of our research group in finding an alternative therapy protocol more predictable (and effective) than the dynamic protocol currently used in clinic. The effects of the acoustic patterns on the produced thermal profiles should be deeply and systematically demonstrated for different real transducers to understand these interactions and to apply this knowledge for proposing a novel therapy planning that would account for the transducer particular beam.

In accordance with the results shown in Figures 7 [Fig fig8]–9, the thermal distribution obtained with the ideal acoustic field differed from the experimental thermal distribution mainly in the first 6 cm, which is the region where the major therapeutic effects are expected. Those variations are mainly produced by the differences in the acoustic distributions at the near-field among the model and the experiment, which can be produced by nonuniform vibrations due to transducer radial resonances and layer defects. For the model, the intensity shown in Figure 7(b) is uniformly distributed along depth with larger peaks just on the propagation axis. This average distribution produces temperature increments along depth dependent on the media attenuation coefficient. However, for the experimental situation shown in Figure 7(a), there is an energy concentration at the first 6 cm, and then, the most significant thermal increments are produced at that zone. As shown in Figure 6, the average intensity in both cases decreases with depth in accordance with attenuation. These average intensities were calculated to observe the behavior of the intensity distribution along depth, which could be a possible explanation of the energy concentration observed in the thermal measurements near the transducer; however, both the average intensity plots follow the same tendency and from this representation, we were not able to conclude that the differences in thermal distributions when *z* < 6 cm were produced by a *z*-dependent energy concentration. It can be observed in measurements that heat was concentrated near the transducer, while in the model it decreased exponentially as a function of depth. Patient injuries and ineffective treatments can be the result of these unexpected thermal distributions. Obtaining more accurate thermal distributions should be done by properly modeling the acoustic field of real ultrasound radiators. Because acoustic field models for this kind of applicators adequate for heat generation have not been proposed yet, temperature predictions under real working conditions cannot be accurately made. One possible approach for obtaining more congruent acoustic field was proposed by the present authors in a previous work [1], but this approach has not been applied yet to produce thermal distributions congruent with the experiments. Some adaptations in this proposed acoustic model are necessary since the modeled field still differs in specific regions into the treatment zone, mainly close to the radiator.

Treatment protocols used in ultrasonic physiotherapy often consider that the acoustic field is naturally nonuniform and it is required to apply some actions to reduce the effect of these nonuniformities, for example, the transducer circular movement during the therapy. However, the effects of these real acoustic patterns in the temperature distributions are rarely determined, and under the experimental conditions used for this analysis, real thermal profiles can have up to 15% (and even more for some other transducers) of deviations from the theory. As many physiotherapists use a dynamic protocol to apply the therapy, these experiments should be repeated for this kind of protocols to validate the piston model for studying the therapy under these other conditions. After analyzing our results, it is evident that more realistic acoustic field models are required to get more congruent thermal distributions during ultrasonic physiotherapy. These acoustic field models should account more for other parameters than the beam size and beam acoustic power already included; other possible parameters could be, for example, the level of transducer beam convergence and the kind of intensity distribution at the near-field zone based on measurements. The latter parameter depends on the measured acoustic intensity distribution which, as analyzed in this work, is related to the produced thermal distribution. The former parameter was already studied by Johns et al. [3] Better treatment protocols and safer standards could be the result of a thorough study of the acoustic radiation of these ultrasonic applications.

As the ultrasonic energy is applied to real perfused tissues, the effect of blood flow and* in vivo* attenuation should be incorporated into the models. As a first step in this work, the inclusion of blow flow in the model was omitted since the objective was to determine the contribution of the main acoustic variables into the thermal distribution under very controlled and simple conditions. For including blow flow in following steps, there are some approaches that have been proposed in the literature using* ex vivo* animal organs; however, the range of control of these approaches should be analyzed to not include uncertainties in the model. The variability of attenuation among individuals is another matter that should be commented on. For modeling the temperature distributions dependent on the spatial acoustic pressure, the subdomain representing the phantom was configured to have the same measured attenuation coefficient of the experimental phantom, which permits the direct comparison of the model results and the experiments. Moreover, the conclusions drawn can be valid for another attenuation if its effects are considered in both the experiments and the models. Accurate methods to determine attenuation* in vivo* and in real-time could help us in obtaining better models to study the effects during real therapy [42]. However, using a fixed attenuation is still valid to demonstrate the drawn conclusions; including variability of attenuation in the model is beyond the scope of this paper.

## 5. Conclusions

For the modeled thermal distribution presented in this work, the ideal classical approach of the piston-in-a-baffle was used intentionally to evaluate its usefulness for this application. It was evident that this model did not provide an acceptable approximation to the real acoustic field emitted by this kind of applicators, and the modeled and measured thermal distributions differed mainly due to the differences in the acoustic radiation patterns. This was confirmed when using the measured acoustic field as the input variable in the heating model (model B).

This paper has presented evidences that suggest that ideal acoustic field models using pistons are not sufficiently accurate to study the energy deposition of physiotherapy ultrasonic applicators. As a possible approach, the thermal models should be determined after modeling real acoustic profile (validation involved), without simplifying the radiator using a piston source. The thermal profile is tightly connected to the acoustic field which at the same time depends on the vibration distribution on the transducer surface [1]; simplifying the latter with a piston radiator will produce large errors in the determined acoustic field and these errors will carry important variation in the temperature estimation as demonstrated.
